# Impact of Thoracoscopic Pulmonary Vein Isolation on Right Ventricular Function: A Pilot Study

**DOI:** 10.1155/2018/7392435

**Published:** 2018-02-20

**Authors:** Gijs E. De Maat, Yoran M. Hummel, Alberto Pozzoli, Ottavio R. Alfieri, Michiel Rienstra, Yuri Blaauw, Isabelle C. Van Gelder, Massimo A. Mariani

**Affiliations:** ^1^Department of Cardiothoracic Surgery and Cardiology, University Medical Center Groningen, University of Groningen, P.O. Box 30.001, 9700 RB Groningen, Netherlands; ^2^Department of Cardiology, University Medical Center Groningen, University of Groningen, Groningen, Netherlands; ^3^Heart Surgery Unit, San Raffaele University Hospital, Milan, Italy

## Abstract

**Objective:**

Thoracoscopic surgical pulmonary vein isolation (sPVI) has been added to the treatment of atrial fibrillation (AF), showing excellent efficacy outcomes. However, data on right ventricular (RV) function following sPVI has never been studied. Our aim was to investigate RV function following sPVI and compare it to patients who underwent endocardial cryoballoon PVI.

**Methods:**

25 patients underwent sPVI and were pair-matched according to age, sex, and AF type with 21 patients who underwent cryoballoon PVI. RV function was measured using tricuspid annular plane systolic excursion (TAPSE) and RV strain with 2D speckle tracking. Echocardiography was performed at baseline and at median 6-month follow-up.

**Results:**

Age was 54 ± 9 years and 84% were male; AF was paroxysmal in 92%. In the sPVI group, TAPSE was reduced with 31% at follow-up echocardiography (*p* < 0.001) and RV strain showed a 25% reduction compared to baseline (*p* = 0.018). In the control group, TAPSE and RV strain did not change significantly (−3% and +13%, *p* = 0.410 and *p* = 0.148). Change in TAPSE and RV strain was significantly different between groups (*p* ≤ 0.001 and *p* = 0.005).

**Conclusions:**

This study shows that RV function is significantly decreased following sPVI. This effect was not observed in the cryoballoon PVI control group.

## 1. Introduction

In the recent years, thoracoscopic surgical pulmonary vein isolation (sPVI) has been added to the treatment of atrial fibrillation (AF). This technique has been shown to be safe and numerous studies have shown excellent efficacy outcome in paroxysmal and short-standing persistent AF due to high transmurality yielded epicardially by bipolar radiofrequency devices [1, 2]. However, right ventricular (RV) function following sPVI has not been investigated. The occurrence of RV dysfunction is not easily predictable and is often unexpected. However, RV function is a major determinant of clinical outcomes following cardiac surgery [3]. With the development of speckle tracking echocardiography, assessment of the RV has become more accessible and reliable for routine clinical practice [4]. The aim of this study was to investigate the right ventricular (RV) function in patients who underwent sPVI and compare it to cryoballoon PVI outcomes.

## 2. Materials and Methods

We studied a series of patients who underwent sPVI as a first PVI procedure during the period of 2009–2011 in our university medical center. Inclusion criteria were highly symptomatic paroxysmal or early persistent AF, without concomitant cardiac structural disease, refractory to class I and/or class III antiarrhythmic drugs [2]. Exclusion criteria for surgical PVI were left atrial size > 55 mm (parasternal view), prior transcatheter PVI, prior heart or lung surgery, significant coronary disease or previous myocardial infarction, left ventricle hypertrophy > 12 mm, previous hospitalization for heart failure, left ventricular dysfunction (ejection fraction < 50%), moderate or severe mitral or aortic valve disease, or lung disease (prior tuberculosis or chronic obstructive pulmonary disease, GOLD classes III-IV). The control group consisted of patients with the same indication who underwent cryoballoon PVI in the same period, also as first invasive procedure. Patients who underwent sPVI were pair-matched retrospectively according to age, sex, and AF type with patients who underwent cryoballoon PVI. All sPVI patients were treated using the video-assisted bilateral thoracoscopy. To isolate the pulmonary veins, a bipolar radiofrequency clamp (Isolator, AtriCure, Cincinnati, Ohio, USA) was used to create linear, thermal lesions. Following the ablation, exit block was confirmed, no additional linear ablation lines were applied, and the left atrial appendage was not excluded [1, 2]. The lateral incision of the pericardium was not routinely closed after completion of the lesion set. The control group consisted of patients who underwent endocardial PVI using the second-generation cryoballoon (Arctic Front Advance™, Medtronic CryoCath LP, Pointe-Claire, Canada). Following ablation, antiarrhythmic drugs were continued during the first three months. Patients underwent a standardized transthoracic echocardiogram preoperatively and at 6-month (range: 3–12) follow-up. Offline analyses were performed by an expert sonographer (Yoran M. Hummel) who was blinded with respect to treatment type. All measurements were performed using EchoPAC BT12, following the 2015 recommendations of the American Society of Echocardiography [5]. RV function was measured using tricuspid annular plane systolic excursion (TAPSE-M-mode) and RV longitudinal deformation (strain) with 2D speckle tracking; in an apical four-chamber view, the edge of the RV endocardium was manually traced, after which the software automatically generated tracings. The basal, middle, and apical segments of the RV were traced. In the analysis, a mean strain of these three segments was calculated. To reliably measure RV function, patients with AF or atrial flutter during echocardiography were excluded from the analysis.

### 2.1. Statistics

Baseline descriptive statistics are presented as mean ± standard deviation or median (range) for continuous variables, as appropriate, and counts with percentages for categorical variables. Differences between groups, in terms of patient characteristics at baseline and different follow-up moments, were evaluated by Student's* t*-test or Mann–Whitney* U* test, depending on normality of the data. Differences within subgroups were evaluated using the paired* t*-test. Chi-square or Fisher's exact test was used for comparison of categorical variables. The statistical software package IBM SPSS Statistics v.22 was used.

## 3. Results

**Figure 1:**
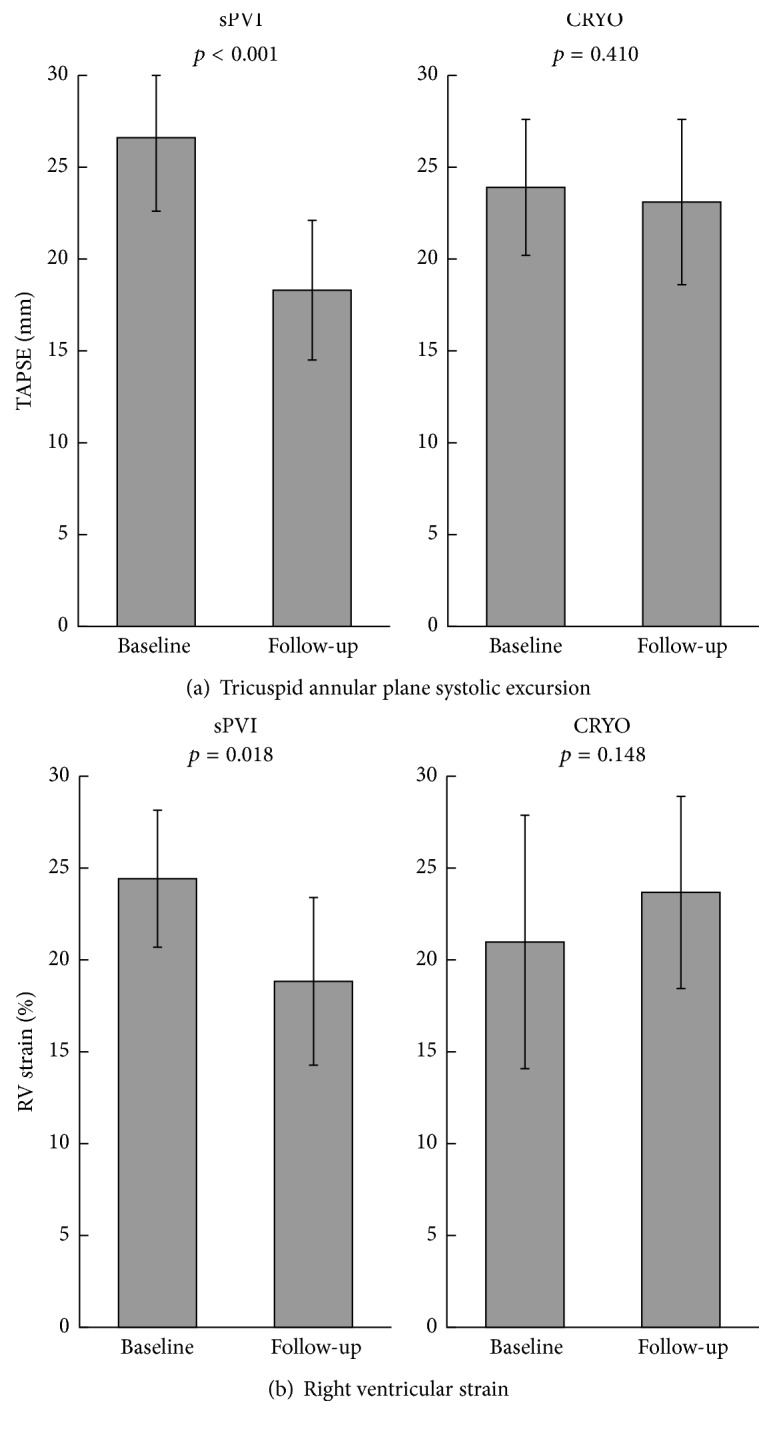


The sPVI group consisted of 25 patients, mean age was 54 ± 9 years, and 84% were male. AF was paroxysmal in 92% and short term persistent in 8%. The control group consisted of 21 patients with similar characteristics who underwent cryoballoon PVI in the same center and period. Baseline patient characteristics did not differ significantly between groups (Table 1). There was no concomitant structural coronary, heart, or valve disease present at baseline. At baseline, echocardiography parameters were comparable between groups except for RA length, which was larger in the sPVI group (57.8 ± 6.2 versus 52.8 ± 5.1, *p* = 0.005), but RA width did not differ between groups (43,7 ± 5,9 versus 41,6 ± 5,1, *p* = 0.212). At baseline echocardiography, TAPSE was higher in the sPVI group compared to the cryoballoon group (26,6 ± 4,0 versus 23,9 ± 3,7, *p* = 0.025; Table 2). In the sPVI group, RV function measured by TAPSE was significantly reduced with a mean of −8.3 mm (−31%) at median 6-month follow-up echocardiography (*p* < 0.001) (Figure 1(a)). Furthermore, the average RV strain showed a mean change of −5.6 percentage points (−25%) compared to baseline echocardiography (*p* = 0.018) (Figure 1(b)). In the control group, the TAPSE was reduced with a mean of −0.8 mm (−3%) and RV strain increased with 2.7 percent points (+13%); this was not significant (*p* = 0.410 and *p* = 0.148, resp.). When the change from baseline to follow-up (delta) measurement in TAPSE and RV strain was compared between the two groups, this showed a significant difference (mean TAPSE −8.3 mm versus −0.8 mm, *p* ≤ 0.001, and mean RV strain −5.6 percentage points versus +2.7 percentage points, *p* = 0.005).

### 3.1. Procedural Outcomes

In all sPVI patients, the procedure was completed with proven acute exit block. Mean procedural time was 160 ± 60 minutes. Mean hospitalization was 7 ± 2 days. There was no 30-day or 1-year mortality. At 12-month follow-up, 88% of sPVI patients were free from atrial arrhythmia and antiarrhythmic drugs. In the cryoballoon group, all patients underwent successful PVI with proven entry and exit block. Mean procedural time was 100 ± 20 minutes. Mean hospitalization was 3 ± 1 days. There was no 30-day or 1-year mortality. At 12-month follow-up, 67% of patients were free from atrial arrhythmia and antiarrhythmic drugs.

## 4. Discussion

This study shows that RV function is significantly decreased following sPVI during the first year. This effect was not observed in our control group, a similar patient population who underwent cryoballoon PVI. Both study groups underwent echocardiographic analysis preoperatively and at 6-month follow-up; there was no significant valve disease and atria were moderately dilated in both groups without significant differences at baseline, except for RA length, which was larger in the sPVI, but RA width did not differ between groups. In both groups, the left atrium was moderately dilated in concordance with the disease. At baseline, mean TAPSE was significantly higher in the sPVI group compared to the CRYO group. However, at follow-up, this was significantly lower. This effect was objectified by means of RV strain.

Decreased RV function following sPVI has not been described previously. On the other hand, in patients who underwent open-chest CABG (both on and off pump), a reduced RV function has been already documented [6]. Remarkably, a study comparing conventional surgical aortic valve replacement (AVR) to transcatheter AVR demonstrated a similar reduction of RV strain at follow-up in patients who underwent surgery [7]. Although the present analysis does not allow definite conclusions regarding the exact underlying mechanism, the reduction of RV function might be attributable to two factors. First, due to the lateral opening of the pericardium, the mechanical support (restraint) is reduced. The right atrium and right ventricle have relatively limited intrinsic stiffness, compared to the left heart side, and are therefore more dependent on pericardial support [8]. Second, the opening of the pericardium causes an inflammatory reaction, which leads to the formation of adhesions between the pericardium and the epicardium. These adhesions can reduce compliance, especially of thin-walled chambers (RA and RV), and thereby impair ventricular filling. Of course, also a combination of both factors could contribute to a reduced RV function following sPVI. Whether decrease in RV function is permanent is associated with symptoms or even heart failure needs to be determined in future studies. An appropriate understanding of pericardial constraint is required. The observational nature of our study and limited number of patients do not allow definitive conclusions.

In conclusion, this study shows that RV function is significantly decreased following sPVI during the first year. This effect was not observed in a similar patient population that underwent cryoballoon PVI. In accordance with the findings of this study, our operative protocol has changed. The right pericardial incision is now routinely closed with approximating endosutures.

## Figures and Tables

**Table 1:** Baseline characteristics and echocardiography outcomes.

Baseline patient characteristics			
Parameter	sPVI (*n* = 25)	CRYO (*n* = 21)	*p* value
Age (years)	54 ± 9	58 ± 7	0.089
Male (*n*, %)	21 (84%)	15 (71%)	0.303
AF history, years [range]	3 [1–14]	4 [1–15]	0.178
Paroxysmal AF (*n*, %)	23 (92%)	17 (81%)	0.268
Short-standing persistent AF (*n*, %)	2 (8%)	4 (19%)	
BMI (kg/m^2^)	27.1 ± 2.9	28.2 ± 3.3	0.251
Systolic BP (mmHg)	132 ± 17	137 ± 16	0.383
Diastolic BP (mmHg)	79 ± 9	83 ± 10	0.234

AF: atrial fibrillation; BMI: body mass index; BP: blood pressure; sPVI: surgical pulmonary vein isolation; TAPSE: tricuspid annular plane systolic excursion.

**Table 2:** Echocardiography outcomes.

sPVI group echocardiography				CRYO group echocardiography		
Parameter	Baseline	Follow-up	*p* value	Baseline	Follow-up	*p* value
LV ejection fraction (%)	60 ± 5	57 ± 5	0.062	60 ± 6	58 ± 7	0.198
LA volume (mm3)	75 ± 19	78,3 ± 23.6	0.674	79 ± 26	71.0 ± 26.0	0.209
LA volume indexed	33,7 ± 6	35,8 ± 10.2	0.430	36.3 ± 11	34.0 ± 12.5	0.414
RA length (mm)	57,8 ± 6.2^*∗*^	57,4 ± 5.3	0.647	52.8 ± 5.1^*∗*^	50.1 ± 5.5	0.048
RA width (mm)	43,7 ± 5.9	43,7 ± 5.1	0.835	41.6 ± 5.1	37.6 ± 5.8	0.035
RVEDD (%)	38,8 ± 5.5	39,0 ± 5.3	0.771	39.6 ± 7.9	37.4 ± 4.5	0.332
TAPSE (mm)	26,6 ± 4.0^*∗*^	18,3 ± 3.8	<0.001	23.9 ± 3.7^*∗*^	23.1 ± 4.5	0.410
RV strain (%)	24.4 ± 3.7	18.8 ± 4.6	0.018	21.0 ± 6.9	23.7 ± 5.2	0.148

LA: left atrial; LV: left ventricular; RA: right atrial; RVEDD: right ventricle end diastolic diameter; sPVI: surgical pulmonary vein isolation; TAPSE: tricuspid annular plane systolic excursion. ^*∗*^Significant difference (*p* < 0.05) between groups at baseline.
